# Functional and Molecular Characterization of Hyposensitive Underactive Bladder Tissue and Urine in Streptozotocin-Induced Diabetic Rat

**DOI:** 10.1371/journal.pone.0102644

**Published:** 2014-07-22

**Authors:** Jayabalan Nirmal, Pradeep Tyagi, Yao-Chi Chuang, Wei-Chia Lee, Naoki Yoshimura, Chao-Cheng Huang, Bharathi Rajaganapathy, Michael B. Chancellor

**Affiliations:** 1 Department of Urology, Kaohsiung Chang Gung Memorial Hospital, Chang Gung University College of Medicine, Kaohsiung, Taiwan; 2 Department of Urology, University of Pittsburgh School of Medicine, Pittsburgh, Pennsylvania, United States of America; 3 Department of Pathology, Kaohsiung Chang Gung Memorial Hospital, Chang Gung University College of Medicine, Kaohsiung, Taiwan; 4 Department of Urology, Centre for Urology Research Excellence, Oakland University William Beaumont School of Medicine, Royal Oak, Michigan, United States of America; University of California Riverside, United States of America

## Abstract

**Background:**

The functional and molecular alterations of nerve growth factor (NGF) and Prostaglandin E2 (PGE2) and its receptors were studied in bladder and urine in streptozotocin (STZ)-induced diabetic rats.

**Methodology/Principal Findings:**

Diabetes mellitus was induced with a single dose of 45 mg/kg STZ Intraperitoneally (i.p) in female Sprague-Dawley rats. Continuous cystometrogram were performed on control rats and STZ treated rats at week 4 or 12 under urethane anesthesia. Bladder was then harvested for histology, expression of EP receptors and NGF by western blotting, PGE2 levels by ELISA, and detection of apoptosis by TUNEL staining. In addition, 4-hr urine was collected from all groups for urine levels of PGE2, and NGF assay. DM induced progressive increase of bladder weight, urine production, intercontraction interval (ICI) and residual urine in a time dependent fashion. Upregulation of Prostaglandin E receptor (EP)1 and EP3 receptors and downregulation of NGF expression, increase in urine NGF and decrease levels of urine PGE2 at week 12 was observed. The decrease in ICI by intravesical instillation of PGE2 was by 51% in control rats and 31.4% in DM group at week 12.

**Conclusions/Significance:**

DM induced hyposensitive underactive bladder which is characterized by increased inflammatory reaction, apoptosis, urine NGF levels, upregulation of EP1 and EP3 receptors and decreased bladder NGF and urine PGE2. The data suggest that EP3 receptor are potential targets in the treatment of diabetes induced underactive bladder.

## Introduction

Diabetes Mellitus (DM), a chronic metabolic condition, is a rising health concern worldwide with significant medical and health economic issues. More than 80% of diabetes patients suffer lower urinary tract complication [1]. Diabetic bladder dysfunction (DBD) is a complex phenomenon and involves different pathophysiology, mechanisms, and time dependent morphological and functional manifestations of bladder [2]. DBD was earlier described as diabetic cystopathy and characteristics include diminished sensation of bladder fullness, reduced bladder contractility and increased residual urine [3], [4], [5].

Recent studies characterize DBD as biphasic with overactive bladder symptoms in the early phase and later phase characterized by hyposensitive and underactive bladder with poor bladder emptying. Later phase complications of DBD leads to chronic urine retention and recurrent urinary tract infections. These functional changes may induce compensatory bladder tissue remodeling such as bladder wall thickening that is promoted by oxidative stress and inflammation in streptozotocin induced diabetic rats [6]. With progression of diabetes, DBD can morph into a state that can be described as detrusor underactivity and hyposensitive bladder [7], [8] or diabetic cystopathy.

Prostanoids, particularly PGE2 are local autocoids and play an important role in micturition reflex. PGE2 synthesis occurs locally in urothelium and smooth muscle [9], [10]. Diabetes impairs Prostaglandin E2 and F2 release from rat urothelium preparations [11]. PGE2 can act directly on the bladder smooth muscle, and also sensitize capsaicin sensitive afferent nerves [12]. In rats and humans, intravesical administration of PGE_2_ stimulates reflex micturition and causes bladder overactivity through EP receptors (EP1–EP4) [12], [13], [14]. The differences in the functions and localization of EP receptors, determine the influence of different EP receptors in micturition processing. EP1 and EP3 receptors expressed in smooth muscle and other tissues[15], [16], [17], [18] are known to cause contraction of non-diseased and cystopathic bladder [19]. However the role of differentially expressed EP1 and EP3 receptors in diabetes induced hyposensitive bladder is not clear.

The diagnosis of diabetic cystopathy relies on urodynamic study, which is invasive and uncomfortable for some patients. A non-invasive biomarker reflecting the different stage and condition of diabetic cystopathy is currently lacking. Nerve Growth Factor (NGF) is produced by both urothelium and smooth muscle in bladder, and believed to be a significant mediator in the modulation of various urothelial responses in diabetic cystopathy [20], [21]. NGF levels decreased in the bladder and L6 and S1 dorsal root ganglion 3 weeks after STZ injection in a time dependent fashion, and the hyposensitive bladder could be reversed by NGF gene therapy at week 8 [22].

In the present study, we investigated the Streptozotocin induced DM rat model at baseline 4 and 12 weeks post STZ injection to study: 1) the time dependent changes in urine biomarkers of NGF, PGE2; 2) differential expression of EP1and EP3 receptors, NGF and PGE2 in control and diabetes (hyposensitive bladder); 3) diabetes induced apoptotic changes. We further studied the effects of intravesical PGE2 in diabetic hyposensitive bladder.

## Materials and Methods

### Animals

Female Sprague-Dawley (SD) rats (220–280 gm), aged 12 to 16 weeks were procured from Bio LASCO Taiwan Co., Ltd. All experimental procedures performed were approved by the Institutional Animal Care and Use Committee of Kaohsiung Chang Gung Memorial Hospital (CGMH 201111183). The rats were acclimated for one week under standardized temperature (21–22°C) and humidity (50–60%) with free access to food and water before the experiment. Streptozotocin (STZ; 45 mg/kg) was injected intraperitoneally to induce DM. Blood glucose levels were measured 5 days after STZ administration. Only rats with blood glucose >350 mg/dl were used in the study. A total of 54 rats were used in this study (N = 18; control group, DM 4 wk, DM 12 wk). Among the 54 rats, 18 rats were used for CMG involving intravesical PGE2 study and 36 rats were used for all other studies.

### Cystometrogram (CMG)

Animals (N = 8, per group) were anesthetized by subcutaneous injection of urethane (1.2 g/kg). A polyethylene tubing (PE-50) was inserted into the bladder through the urethra and connected via a three-way stopcock to a pressure transducer and to a syringe pump for recording intravesical pressure and for infusing solutions into the bladder. To elicit repetitive voiding, CMG was performed by filling the bladder with saline (0.08 ml/min). Intercontraction interval (ICI), the average time between contractions of reflex bladder contractions; amplitude (the peak pressure minus the basal pressure during each contraction period); pressure baseline (PB), the pressure immediately after the reflex contraction); and pressure threshold (PT), the pressure immediately before the reflex contraction were recorded. Measurements in each animal represented the average of 3 to 5 bladder contractions after an initial 60 minute stabilization period. At the end of CMG recording, the saline infusion was stopped after confirming the first micturition, and then the residual urine was collected and measured.

### Urine Cytokines

Urine samples were collected in iced bowls for 4 hr from 9 rats (N = 9) at baseline, 4 week, and 12 week post-STZ injection. The collected urine was centrifuged at 3000 rpm for 10 minutes at 4°C. The supernatants were immediately frozen at −80°C and kept until further analysis. On the day of analysis, frozen urine samples were thawed at room temperature for measurement of urine PGE2 (Cayman, Michigan, USA), NGF (EIAab, Wuhan, China) using ELISA kits for rat urine according to the manufacturer's instructions. The NGF concentrations for each time point were normalized to the creatinine concentration in urine and are expressed as the picograms excreted per milligram of creatinine.

### Western blot analysis for EP1, EP3, and NGF expression

Rats were euthanized in control group and in experimental groups (N = 6, per group) at 4 and 12 weeks post STZ injection. The bladder was weighed and subjected to Western blot analysis of EP1and EP3 receptor expression according to the standard protocol and following our previous study [23]. Bladder was homogenized in protein extraction solution (T-PER; Pierce Biotechnology) prior to sonication and purification. Total protein was measured using Bradford protein assay (Bio-Rad Laboratories, Hercules, CA). Lemmli buffer system was used to run the SDS-polyacrylamide gel electrophoresis (PAGE). Briefly, 30 µg protein aliquot was loaded onto 8% polyacrylamide gel, electrophoresed at a constant voltage of 100 V for 1 h and transferred to Hybond-P PVDF Membrane (Amersham Biosciences). Blocking agent was used to block the membrane and then immunoblotted overnight at 4°C. The primary antibody concentration of mouse anti-actin monoclonal antibody (Chemicon, USA; MAB1501); rabbit anti-NGF polyclonal antibody (Santa Cruz, USA; SC548) and rabbit anti-EP1 and EP3 receptor polyclonal antibody (Cayman, MI, USA; No.101740 and 101760, respectively) were used as follows: Mouse anti-actin (1∶10000); rabbit anti-NGF (1∶1000); rabbit anti-EP1 (1∶200); rabbit anti-EP3 (1∶500). The molecular weight of NGF, EP1 receptor, and EP3 receptor is 27 kDa, 42 kDa and 52 kDa, respectively as per the manufacturer. Washed membranes were incubated with secondary antibody using 5% defatted milk powder in Tris buffered saline TBS for 2 h at room temperature using a horseradish peroxidase-linked anti-rabbit or anti-mouse Immuglobulin G. Visualized the Western blots using enhanced chemiluminescence (ECL) detection system (Amersham Biosciences). β-actin was used as the internal control. Quantitative analysis was done using LabWorks Image Acquisition and Analysis software.

### ELISA analysis of PGE2

Enzyme immunoassay kits (Cayman Chemical, Ann Arbor, MI, USA, Catalog No.514010) were used to measure levels of PGE2 in rat bladder according to the directions of manufacturer. In brief, frozen rat bladder was homogenized in 0.1 mol/L phosphate-buffered saline with 1 mmol/L EDTA and 10 mmol/L indomethacin was added. Homogenization samples were suspended in ethanol (4 times of sample volume) and incubated for 5 min, and then samples were centrifuged at 3000 g at 4°C for 10 min. The supernatant was transferred into a clean test tube cautiously. In enzyme immunoassay buffer the resulting samples were re-dissolved and transferred to an antibody precoated 96-well plate. Total Activity (TA), Non Specific Binding (NSB) and substrate blank wells for comparison were marked in antibody-coated wells. In to the zero standard (B0; 50 µL) and NSB (100 µL) wells, assay buffer (a buffered protein base) was added. 50 µL of diluted samples and PG standards were pipetted into the residual wells. Conjugate of high-sensitivity (50 µL) was added to each well, apart from the TA and substrate blank wells. Solution of High-sensitivity antibody (50 µL) was dispensed into every well, not including the NSB, TA and substrate blank wells. Adhesive strips were used to cover the wells till reaction. Washing was performed 5 times with washing buffer and then all liquid was removed from the wells. Five ml of conjugate was added to TA well and 200 µL of Ellman's reagent was added to each well. PGE2 concentration was determinedd after absorbance measurement at 405 nm with a microplate reader (BIO-TEK, μQuant, USA) [24].

### Histology and immunohistochemistry

After the CMG study, 6 animals from each group were deeply anesthetized with pentobarbital and sacrificed via transcardiac perfusion, first with Krebs buffer followed by 4% paraformaldehyde fixative. Harvested bladder was weighed and divided into two parts. One part was fixed in 10% buffered formaldehyde for 24–48 h, and then embedded in paraffin, and stained with hematoxyline and eosin or subjected to immunohistochemistry analysis for apoptosis with TUNEL stain (terminal deoxynucleotidyl-mediated deoxyuridine triphosphate nick end labeling stain) using a commercially available kit (Detection Kit POD, Boehringer Mannhein, Mannhein, Germany). Remaining part of bladder was frozen in liquid nitrogen and stored at −80°C until use. Inflammation associated with STZ induced cystopathy was graded by a score of 0–3 as follows: 0- no evidence of inflammatory cell infiltrates or interstitial edema; 1- mild (few inflammatory cell infiltrates and little interstitial edema); 2- moderate (moderate amount of inflammatory cell infiltrates and moderate interstitial edema); 3- severe (diffuse presence of a large amount of inflammatory cell infiltrates and severe interstitial edema). A single blinded research technician graded the bladder histology. The proportion of TUNEL positive nuclei per 500 nuclei was quantified at ×400 magnification using a 10×10 grid in the eyepiece in 4 fields.

### Intravesical PGE_2_ administration

Stock solutions (0.01 M) of PGE_2_ (Sigma) were made in absolute ethanol and then further diluted in saline (100 µM) before use. After a baseline measurement was established during saline infusion, 100 µM PGE2 was infused into the bladder (N = 6, per group) at 0.08 ml/min to acutely simulate bladder hyperactivity. The CMG was performed in control and STZ rats at week four and week 12 to test the response of DM rats to intravesical PGE2.

### Statistical analysis

Quantitative data are expressed as mean ± standard error. Statistical analyses were performed using t-test or one way ANOVA, with Dunn's multiple comparisons test where applicable, with p<0.05 considered significant.

## Results

### DM induced physiological changes

Body weight of diabetic rats decreased by the 4^th^ and 12^th^ week following STZ injection. The decrease was found to be significant at week 4 compared to the control group. At week 12 a significant increase in body weight was noted as compared to week 4. Significantly increased bladder weight was observed till 12 weeks. Significant rise in the blood glucose and urine production was noted compared to control group. Progressive increase of bladder weight, urine production is considered signs for developing DBD (Table 1).

**Table 1:** DM induced change of body weight, bladder weight, serum glucose level, and urine production.

N = 12 Per group (SEM)	Body Weight (gm; Initial)	Body Weight (gm; Final)	Body weight change (gm)	Bladder Weight (gm)	Bladder Weight/Bodyweight (gm/100 gm)	Blood Glucose (mg/dl; Initial)	Blood Glucose (mg/dl; Final)	Urine/hr (cc)
Control	301.58(7.66)	309.58(9.94)	8.00(5.64)	0.15(0.01)	0.05(0.00)	109.00(5.90)	102.00(6.61)	0.61(0.10)
DM4 wk	303.92(4.43)	266.00*(7.58)	37.92**(8.21)	0.34**(0.01)	0.13**(0.00)	115.67(4.59)	531.92**(27.67)	8.19**(0.94)
DM 12 wk	300.50(7.68)	289.00(3.35)	11.50@(6.89)	0.48** @@(0.02)	0.17** @@(0.01)	102.33(4.12)	546.00**(15.44)	11.30**(0.86)

*P<0.05 vs control;

**P<0.0001 vs control;

@ P<0.05 vs 4 wk;

@@ P<0.0001 vs 4 wk.

### DM induced cystometry changes

**Figure 1:**
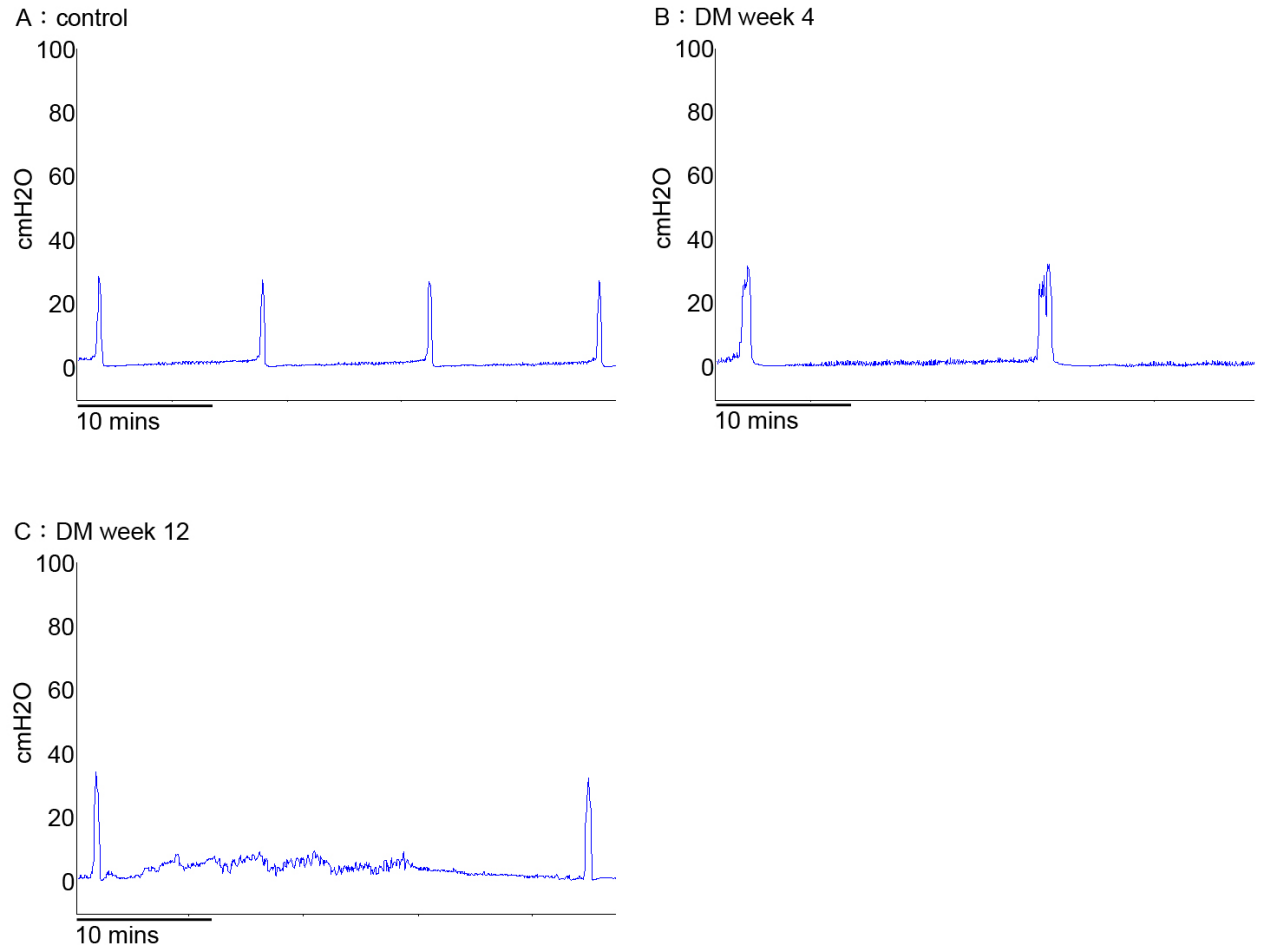
Representative traces of continuous cystometrograms (CMG) in urethane anesthetized rats (N = 8 per group; A- Control, B- DM week 4 and C- DM week 12). A with a normal voiding pattern, B and C with a hyposensitive underactive bladder.

CMG analysis showed an increased ICI at week 4 and 12 when compared to the control group. Time dependent increase in ICI (2.1 fold and 2.6 fold increase) and residual urine (2.7 and 2.9 ml) at week 4 and 12 confirms the development of hyposensitive underactive bladder due to DM (Table 2; Fig. 1). In the current study, we did not observe overflow incontinence from the control rats as well as the DM rats. Non-voiding contractions were observed in some DM rats (Fig. 1 C).

**Table 2:** Cystometric analysis of the experimental groups.

N = 8 per group (SEM)	ICI, min	Amp, cmH_2_O	BP, cmH_2_O	PT, cmH_2_O	RU(gm)
Control	11.3(0.1)	26.2(1.7)	1.1(0.2)	4.1(0.5)	0.1(0.1)
DM 4 wk	23.8(2.9)*	30.4(1.6)	1.3(0.2)	3.9(0.4)	2.7(0.4)**
DM 12 wk	29.9(2.6)**	29.1(1.8)	1.1(0.2)	4.2(0.4)	2.9(0.7)**

ICI: intercontraction interval; Amp: amplitude; BP: baseline pressure; PT: pressure threshold; RU: Residual urine.

*P<0.05 vs control;

**P<0.01 vs control.

### DM induced changes in urine markers

Diabetic cystopathy gradually decreased the PGE2 levels in the urine at week 4 (12.5% decrease) and at week 12 (70.5% decrease) as compared to the baseline levels. The decrease of urinary PGE2 was significant at week 12. The urine NGF level was progressively increased at week 4 (5.4 fold increase) and week 12 (6.25 fold increase) as compared to baseline (Table 3).

**Table 3:** Analysis of urine cytokines in DM rats.

N = 9 (SEM)	PGE2/creatinine (pg/µg)	NGF/creatinine (pg/µg)
Baseline	25.49 (9.21)	0.52 (0.07)
DM 4 wk	22.31 (9.01)	2.83 (0.45)*
DM 12 wk	7.53 (0.86)*	3.25 (0.54)*

*P<0.05 vs baseline.

### DM induced changes in the bladder expression of EP receptors, NGF and PGE2

**Figure 2:**
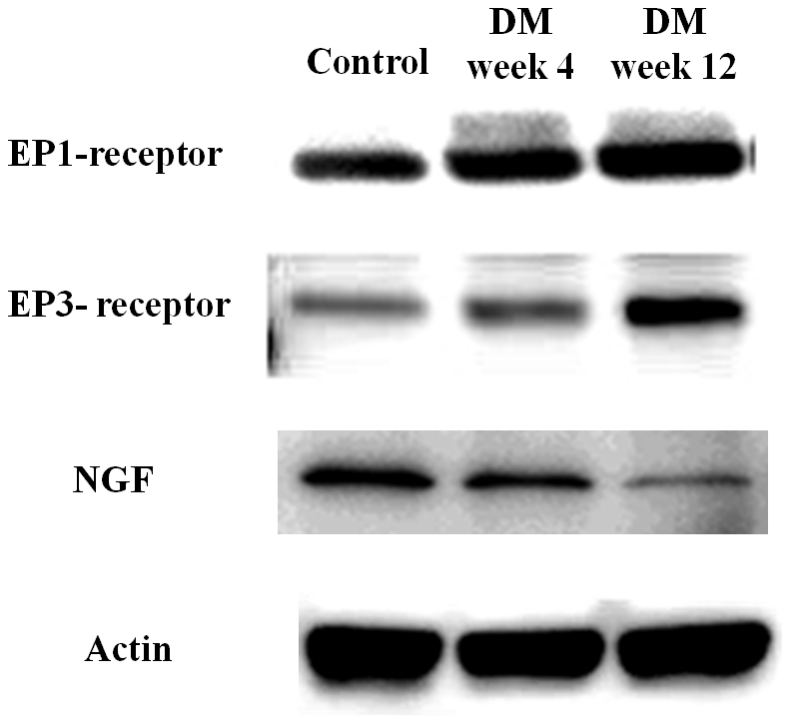
Western blot analysis of EP1, EP3 receptors and NGF expression in the bladder (N = 6, per group). At DM week 12, the protein amount of EP3 was significantly increased (1.4 fold) and NGF was significantly decreased (60.6%) than the control rat. There was no significant change of EP1 receptors.

Western blot analysis relative to anti-actin demonstrated the upregulation of EP1 and EP3 receptors especially 1.4 fold upregulation of EP3 receptor was observed at week 12. NGF level was found to be significantly down regulated (60.6%) at DM week 12 (Table 4 and fig. 2). Expression levels of PGE2 also showed an upregulation at DM week 4 and week 12, however, it was not significant.

**Table 4:** Bladder histology, western blotting for detection of EP1, EP3, NGF, and ELISA for detection of PGE2.

N = 6, Per group (SEM)	EP1	EP3	NGF	PGE2
Control	1.00 (0.00)	1.00 (0.00)	1.00 (0.00)	1.00 (0.00)
DM 4week	1.09 (0.15)	1.34 (0.17)	0.47 (0.10)*	1.71 (0.57)
DM 12week	1.29 (0.16)	1.43 (0.07)*	0.42 (0.10)**	1.31 (0.46)

*P<0.05, and

**P<0.01 vs Control.

### Histology and TUNEL staining

**Figure 3:**
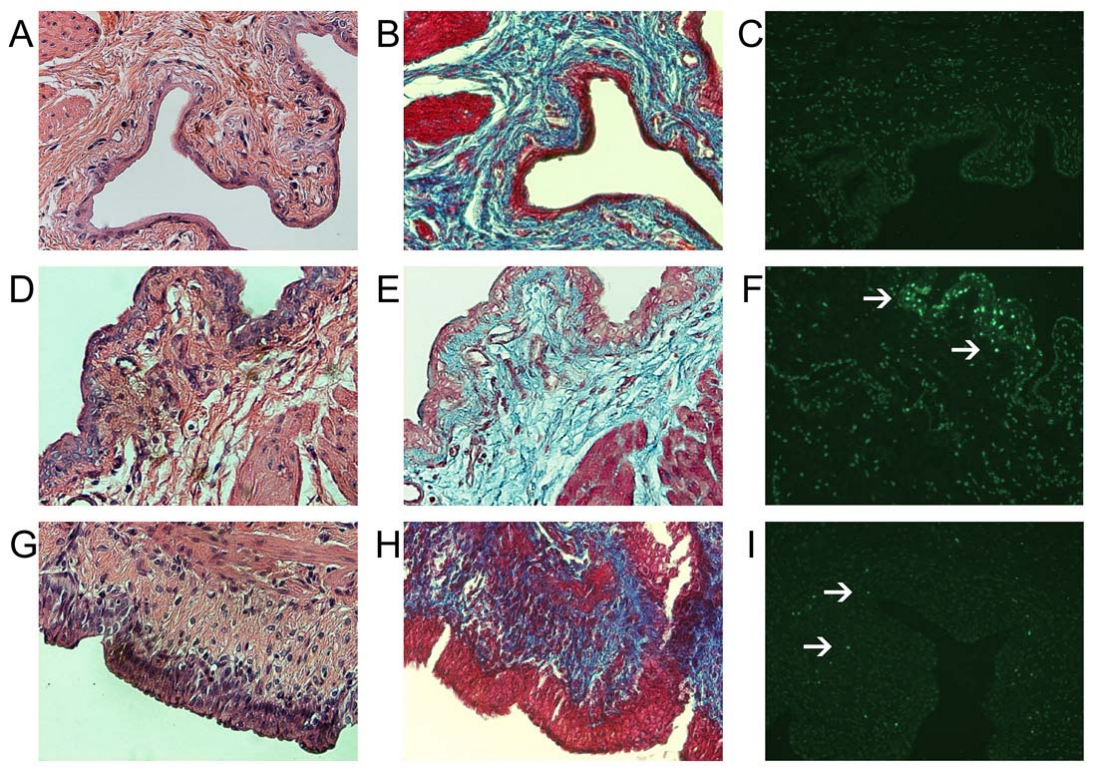
Photomicrographs of bladder sections (N = 6, per group). A, B, C: Control Group; D, E, F: DM week 4; G, H, I: DM week 12, (×200). A, D, G (H and E stain); B, E, H (trichome stain); CFI (TUNEL stain). DM rat at week 12 (G, H) showed increase of inflammatory reaction compared to the control rat (A, B). DM rat at week 4 (F) showed significant increase of TUNEL stain (9.4 fold) compared to the control rat (C).

DM bladder sections showed thickening of submucosal layer and increased inflammatory cell infiltrates (Fig. 3D, G) as compared to the control (Fig. 3A). There was a trend of increased percentage of trichome staining at week 12 which was indicative of inflammation (Fig. 3B, E, H). The increased apoptosis was shown by TUNEL staining in DM group as compared to control (Table 5 and Fig. 3).

**Table 5:** TUNEL assay and histology scores in rat bladder.

N = 6 per group (SEM)	TUNEL Staining	Trichrome stain, percentage	Edema score	Inflammatory Cells
Control	2.70 (0.80)	31.77 (6.04)	0.43 (0.30)	0.32 (0.05)
DM 4 wk	25.40 (5.00)*	32.50 (4.08)	1.57 (0.43)	1.73 (0.21)**
DM 12 wk	17.00 (2.90)	46.10 (4.15)	2.0 (0.44)*	2.2 (0.21)***

*P<0.05 and

**P<0.01 and

***P<0.0001 VS Control.

### Intravesical PGE2 (100 µM) on CMG

**Figure 4:**
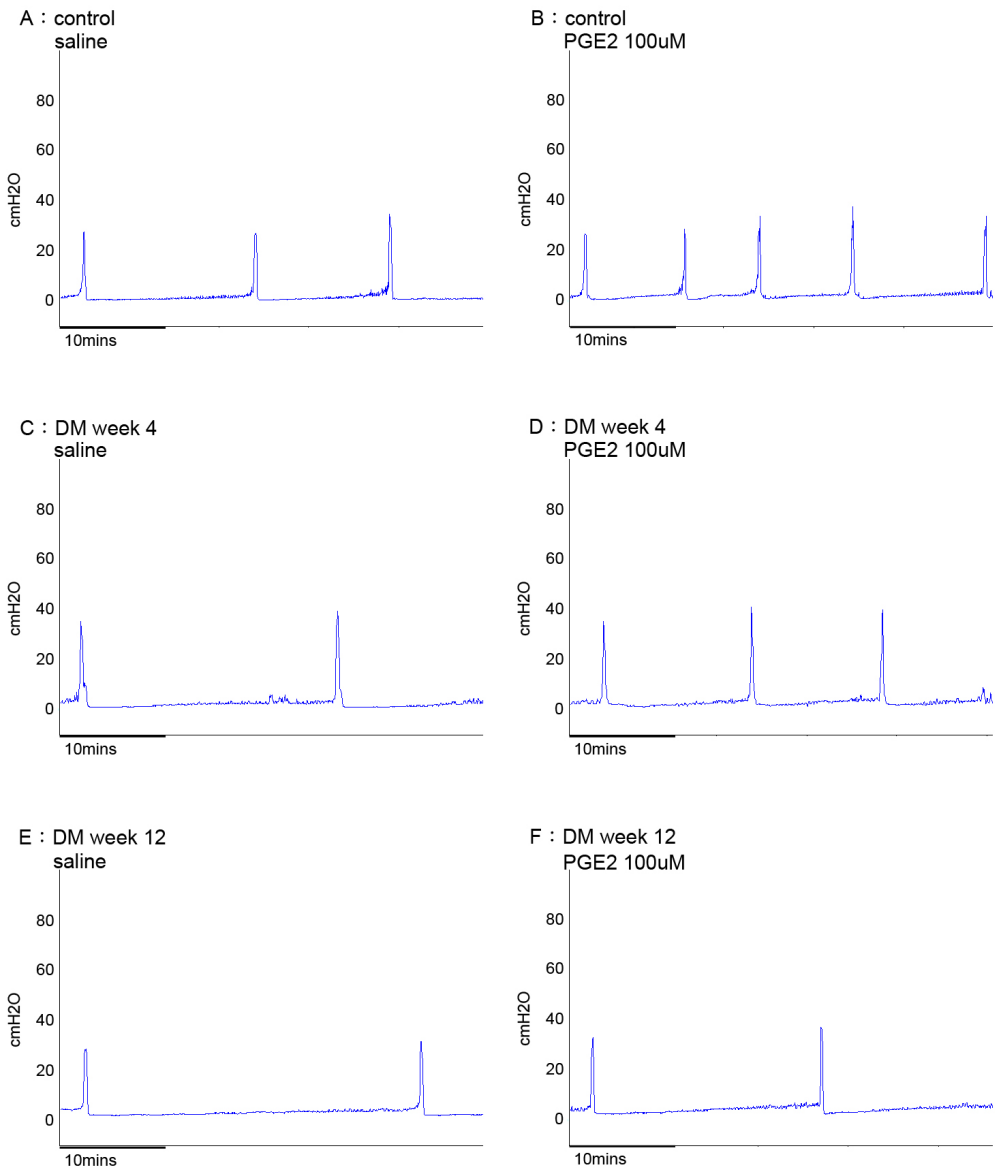
Representative traces of continuous cystometrograms (CMG) in urethane anesthetized rats (N = 6, per group). Intravesical PGE2 induced bladder overactivity, which effects were decreased at DM week 4 and week 12. A (Control, saline), B (Control, PGE2-100 µg); C (DM week 4, saline), D (DM week 4, PGE2 – 100 µg); E (DM week 12, saline) F (DM week 12, PGE2).

ICI was significantly decreased after intravesical instillation of PGE2 (51% decrease) in the control rats. However, rats at 12weeks post –STZ injection showed a tendency to reduce response (ICI 31.4% decrease; p = 0.0849, vs control) to PGE2 instillation. (Table 6; Fig. 4).

**Table 6:** Effects of intravesical PGE2 on CMG parameters.

N = 6 per group (SEM)	Treatment	ICI (min)	Amp (cmH2O)	PB (cmH2O)	PT (cmH2O)
Control	Saline	14.1 (1.4)	26.6 (2.7)	0.9 (0.3)	3.5 (0.3)
	PGE2 100 uM	6.9 (1.0)**	25.8 (2.0)	2.1 (0.4)*	5.7 (0.4)**
DM 4 W	Saline	22.3 (3.1)	30.2 (2.4)	0.8 (0.2)	3.1 (0.3)
	PGE2 100 uM	14.0 (1.9)*	31.1 (3.3)	1.9 (0.5)	4.2 (0.4)
DM 12 W	Saline	31.9 (3.4)#	26.9 (1.6)	1.3 (0.4)	4.2 (0.4)
	PGE2 100 uM	21.9 (5.3)	28.1 (2.0)	2.4 (0.5)	5.4 (0.4)*

ICI: intercontraction interval; Amp: amplitude; BP: baseline pressure; PT: pressure threshold;

*P<0.05 and

**P<0.01;

# P<0.01 (control vs DM 12 W).

## Discussion

Diabetes is associated with a systemic inflammation, neuropathy, and metabolic syndrome, all of which have been linked to DBD [25]. Pathophysiology of diabetic cystopathy is multifactorial and may result from physiological changes in the detrusor smooth muscle cell, the innervation, or dysfunction of urothelium, however, the underlying mechanism is not known [6]. The observation of hyposensitive bladder at week 12 post STZ injection implicates dysregulated paracrine signaling and remodeling of bladder tissue that is characteristic of diabetic cystopathy [1]. Cystometric results (Fig. 1C) are consistent with increased bladder capacity associated with hyposensitive underactive bladder.

The STZ induced diabetes is confirmed in the present study by increase in blood glucose and urine production. Increased bladder weight and residual urine indicate the incomplete bladder emptying in the animals. Increase in the bladder weight of STZ induced DM rats may results from edematous change in the hyposensitive distended bladder. The role of prostaglandins in maintaining the barrier properties and low osmotic water permeability is known [26]. The upregulation of PGE2 and EP1 and EP3 receptors in our study might indicate an adaptive response to decrease osmotic water permeability [26] and counter the edematous changes.

The increase of ICI and residual urine without an accompanying decrease of the contraction amplitude in these observations mimic the situation of hyposensitive bladder (Table 1, Fig. 1). The hyposensitive bladder may arise from compromised nerve function, which may occur earlier than impairment of muscle contractility as neurons are more sensitive to hyperglycemia than muscles [2]. Sensory and sympathetic neurons require NGF for survival and maintenance [21]. The significant decrease in the expression of NGF in diabetic bladder is correlated with the progression of the underactive bladder at week 12 (Table 4). It was postulated that impaired functions of A-δ and C-fiber bladder afferent pathways in diabetic cystopathy is associated with the progressive decline in NGF [27]. On the contrary, NGF over expression in the bladder and bladder afferent pathways is involved in the emergence of hyperexcitability in bladder C-fiber sensory pathways [28], [29]. Previous studies show that down regulated NGF expression in the urinary bladder of diabetic rats leads to its decreased transport to their afferent pathways, which contributes to diabetic cystopathy [22], [30].

Interestingly, we found increased urine levels of NGF together with lowered bladder NGF levels, which may be related to the inflammation, edema and apoptosis in hyposensitive bladder (Table 5; Fig. 3). It has been suggested that NGF is required for the maintenance of the neurons, the ability to resist apoptosis and regenerative capacity [30]. Previous studies have reported that there is a deficiency of NGF in diabetes [26], [30], which is compatible with the current finding of decreased bladder NGF level in our DM rats. Furthermore, inflammation and apoptosis in bladder might cause the loss of NGF binding receptors, which leads to increased expulsion of NGF in bladder and increased urine NGF level. Sasaki et al. has proved the concept that NGF gene therapy could restore the bladder NGF levels and rescue the bladder function in STZ induced diabetic cystopathy [22]. Cyanidin-3-O-b-D-glucopyranoside (C3G), a free radical scavenger to attenuate oxidate stress, has been shown to restore the bladder function in STZ induced DBD by decrease in apoptosis and increase in the NGF bladder levels [31], [32], [33]. However, the change of urine NGF levels were not reported in conjunction with the recovery of bladder function. It would be interesting to know whether the urine NGF level could serve as a surrogate marker to monitor DBD.

Decreased urine levels of PGE2 may be related to loss of cells secreting PGE2 in hyposensitive diabetic bladder epithelium, which is known to impair voiding function and lead to urinary retention [12], [13]. It is known that prostanoid is synthesized locally from both bladder smooth muscle and urothelium [9] and PGE2 modulates bladder function at detrusor muscle as well as efferent and capsaicin sensitive afferent nerves [13], [34]. The deficiency of bladder NGF in DM rats leads to impairment of capsaicin sensitive afferent nerve [26], which tends to a decrease of response to intravesical PGE2 instillation. The present study also aimed to delineate the association between PGE2 and the contraction receptors EP1 and EP3 to develop a therapeutic target for underactive bladder. The expression of EP1 and EP3 receptors in control and upregulation of EP1 and EP3 receptors in the hyposensitive bladder indicates the role of PGE2 in micturition function might partly mediate through these 2 receptors (Table 4; Fig. 2). The upregulation of EP1 and EP3 receptors in DBD may be compensatory changes to maintain bladder contractility [35], [36]. DBD is also known to cause alteration in expression of cannabinoid and TRPV1 receptors in bladder and urinary tract [37], [38].

EP1 and EP3 are stimulatory receptors, mediating smooth muscle contraction by an increase of phosphoinositol turnover and calcium mobilization, or a decrease of adenylate cyclase and cAMP levels, respectively [39], [40]. In guinea pig uterus, PGE2 was shown to be agonists for both EP1 and/or EP3 receptors, but was mostly acting via EP3 receptors [18]. Further studies are needed to clarify the precise link between PGE2 and EP1 and EP3 receptors with regard to the functional changes in urothelium and detrusor in the DM hyposensitive bladder. Intravesical PGE2 induced a decrease in the inter contraction interval (Table 6; Fig. 4) at DM week 4 and 12, which may be caused by direct action on the smooth muscle and activation of capsaicin sensitive afferent pathway leading to phasic contractions [13]. DM is known to lead loss of phasic contractions in bladder due to changes in non-adrenergic noncholinergic transmission and intravesical PGE2 may be able to restore it [41].

In conclusion, we observed inflammation, apoptosis, and upregulation of EP1 and EP3 receptors in DM hyposensitive underactive bladder. Furthermore, changes in the expression of PGE2 and NGF were also noted, whose altered urine levels can serve as surrogate for non-invasively tracking the progressive DBD. We also tested the effect of a potential treatment with intravesical PGE2 administration, which exerts contraction of underactive bladder utilizing EP receptors. Further study targeting at EP3 receptor would be worthy of a trial to identify a potential therapeutic modality for diabetes induced hyposensitive underactive bladder.
